# Chronic granulomatous disease secondary to a rare compound heterozygote mutation in an adolescent cured by hematopoietic stem cell transplantation: a case report

**DOI:** 10.3389/fped.2026.1780075

**Published:** 2026-07-14

**Authors:** Menglan Zhou, Ying Zhao, Xin Sun, Wenjun Mou, Yaping Liu, Chuan Shi, Zongru Li, Yifei Cheng, Xinlun Tian, Junping Fan, Jinglan Wang

**Affiliations:** 1 State Key Laboratory of Complex Severe and Rare Diseases, Department of Laboratory Medicine, Peking Union Medical College Hospital, Chinese Academy of Medical Science and Peking Union Medical College, Beijing, China; 2 Beijing Key Laboratory for Mechanisms Research and Precision Diagnosis of Invasive Fungal Diseases, Beijing, China; 3 Department of Internal Medicine, Peking Union Medical College Hospital, Chinese Academy of Medical Science and Peking Union Medical College, Beijing, China; 4 Laboratory of Tumor Immunology, Beijing Pediatric Research Institute, Beijing Children's Hospital, Capital Medical University, National Center for Children's Health, Beijing, China; 5 Center for Rare Diseases, The State Key Laboratory for Complex, Severe, and Rare Diseases, Peking Union Medical College Hospital, Chinese Academy of Medical Sciences, Peking Union Medical College, Beijing, China; 6 Department of Pulmonary and Critical Care Medicine, Peking Union Medical College Hospital, Chinese Academy of Medical Science and Peking Union Medical College, Beijing, China; 7 National Clinical Research Center for Hematologic Disease, Beijing Key Laboratory of Hematopoietic Stem Cell Transplantation, Peking University Institute of Hematology, Peking University People's Hospital, Beijing, China

**Keywords:** *Burkholderia multivorans*, chronic granulomatous disease, *CYBA* gene, pneumonia, whole exome sequencing

## Abstract

**Background:**

Chronic granulomatous disease (CGD) is a rare inherited primary immunodeficiency characterized by recurrent infections and aberrant inflammation due to defects in the nicotinamide adenine dinucleotide phosphate (NADPH) oxidase complex.

**Case presentation:**

We report a case of recurrent pneumonia and significantly elevated IgE levels in an adolescent. Metagenomic next-generation (mNGS) sequencing contributed to the identification of *Burkholderia multivorans* in bronchoalveolar lavage fluid and the initiation of appropriate treatment. Whole exome sequencing (WES) revealed two point mutations in the *CYBA* gene. The patient was cured by hematopoietic stem cell transplantation.

**Conclusions:**

Application of mNGS contributed to the early identification of *B. multivorans* and the initiation of appropriate treatment. Timely screening by WES contributed to the diagnosis of the patient.

## Background

Chronic granulomatous disease (CGD) is a primary immunodeficiency with defects in the nicotinamide adenine dinucleotide phosphate (NADPH) oxidase complex, resulting in compromised respiratory burst necessary for normal killing of microorganisms by phagocytes (1). This defect is caused by mutations in the genes encoding the NADPH oxidase subunits, including *CYBB* (gp91*^phox^*), *CYBA* (p22*^phox^*), *NCF1* (p47*^phox^*), *NCF2* (p67*^phox^*), and *NCF4* (p40*^phox^*) (1). Pathogenic variants in the *CYBB* gene are inherited in an autosomal recessive manner, while other genes manifest as an autosomal recessive trait (2).

While autoinflammatory manifestations and peripheral infections are hallmarks of this condition, recent reports confirm that severe invasive infections, such as hepatosplenic abscesses, can also occur, underscoring its phenotypic spectrum (3). Furthermore, the genetic landscape of CGD has expanded to include defects beyond the core NADPH oxidase complex. For instance, homozygous loss-of-function mutations in *CYBC1* (also known as *EROS*), a chaperone critical for the stability of gp91phox, result in an autosomal recessive form of CGD with a clinical phenotype akin to classic *CYBB*-deficiency (4).

CGD is often accompanied by recurrent, even life-threatening bacterial and fungal infections, with the most common pathogens including *Staphylococcus aureus*, *Serratia marcescens*, *Burkholderia cepacia* complex, *Nocardia* spp., and *Aspergillus* species (5). In patients with CGD, infections trigger exaggerated or dysregulated inflammatory cascades, which can be associated with immune abnormalities such as hypergammaglobulinemia (6) or less commonly, hypogammaglobulinemia (7).

Although infections with *B. cepacia* complex have been commonly reported in patients with CGD, infections due to *B. multivorans* have rarely been reported, even in immunocompetent patients (8, 9). Herein, we report an adolescent with CGD who developed a *B. multivorans* infection along with significantly elevated immunoglobulin E (IgE) levels.

## Case presentation

**Figure 1:**
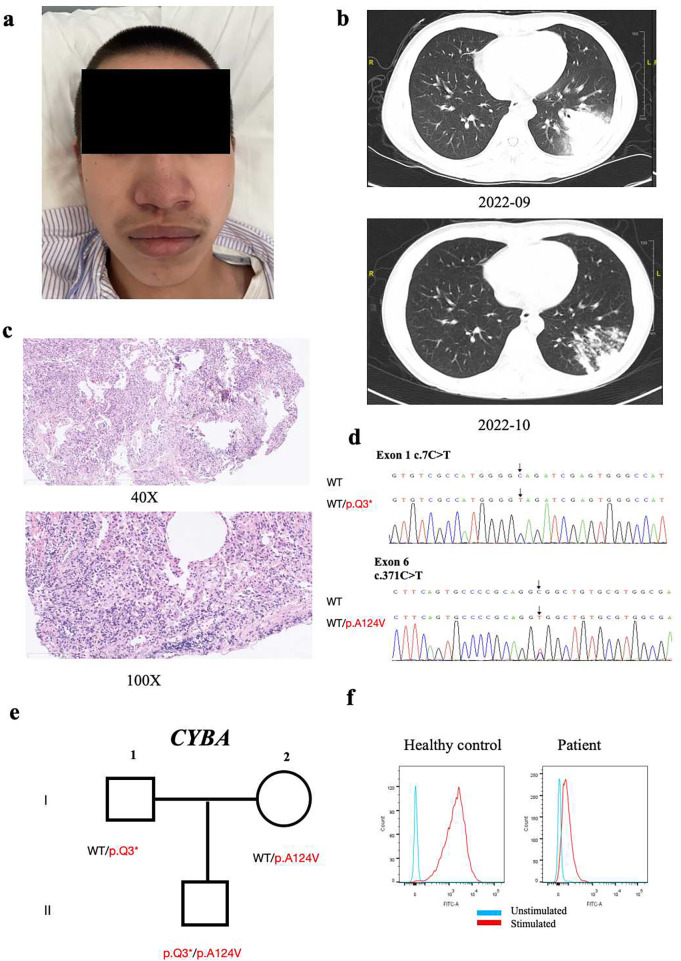
Genetic and clinical features of the patient with a rare compound heterozygote mutation in the *CYBA* gene. **(a)** Facial image of the patient characterized by a wide and thick nose and a full lower lip. **(b)** Comparison of the thorax CT scan images on admission and before discharge, revealing a much smaller size of consolidation in the left lower lobe. **(c)** Transbronchial lung biopsy of the basal segment in the left lower lobe showing chronic inflammation accompanied with focal neutrophil infiltration. **(d)** Electropherogram of exons 1 and 6 showing the variants (p.Q3* and p.A124 V) found in the patient compared with the healthy control. **(e)** Pedigree of the family, showing familial segregation of the *CYBA* gene alleles. Generations are indicated by Roman numerals (I–II), and each individual is indicated by an Arabic numeral (1, 2). **(f)** Dihydrorhodamine assay of the patient and the healthy control.

A 16-year-old boy was referred to this hospital with a two-week history of persistent fever, chills, cough and scant sticky phlegm despite intravenous azithromycin and cefuroxime. Fever (T_max_ 39.5 °C) persisted after escalation of antibiotics to ertapenem and linezolid for six days. Physical examination revealed a normal body mass index (20 kg/m^2^, z-score of −0.18) and distinctive facial features characterized by a wide and thick nose and a full lower lip (Figure 1a). The patient's height (173 cm) and weight (60 kg) were both around the 50th percentile, and pubertal development was appropriate for age (Tanner stage IV).

On admission, leukocytosis, elevated C-reactive protein, erythrocyte sedimentation rate, IgA and IgE were noted. Specific IgE showed moderate allergy to *Aspergillus fumigatus* and mold mix. Galactomannan test, cryptococcus antigen test, Widal test, *Chlamydia* antibody, *Mycoplasma* antibody, *Legionella* antibody, hepatitis panel (HBsAg, HBeAg, anti-HBs, Anti-HBe, Anti-HBc and HCV), parvovirus B19 antibody, cytomegalovirus DNA, Epstein–Barr virus DNA, sputum culture and a total of three sets of peripheral blood culture were all negative (Table 1). Chest CT revealed left lower lobe consolidation suggestive of pneumonia (Figure 1b).

**Table 1:** Laboratory findings of the patient with a rare compound heterozygote mutation in the CYBA gene on admission.

Variable	Detection value	Reference range
White blood cell count (× 10^9^/L)	11.8	3.5–9.5
Platelet count (× 10^9^/L)	229	100–350
Neutrophils (%)	80	50–75
Eosinophils (%)	0.5	0.5–5
C-reactive protein (mg/L)	77.1	≤ 8
Procalcitonin (ng/mL)	< 0.072	-
Erythrocyte sedimentation rate (mm/hour)	32	0–15
Immunoglobulin G	15.95	7–17
Immunoglobulin A	8.17	0.7–4
Immunoglobulin M	1.02	0.4–2.3
Immunoglobulin E	> 5,000	0–60

He had two prior admissions for pneumonia in the preceding six months, which responded to antibiotics including azithromycin, cephalosporin, piperacillin-tazobactam and meropenem. His past medical history included two acute flares of eczema per year between 3 and 10 years of age. Oral ulcers occurred frequently ages 8–14 years without genital involvement. He also developed seasonal rhinitis. No specific family history was reported.

**Figure 2:**
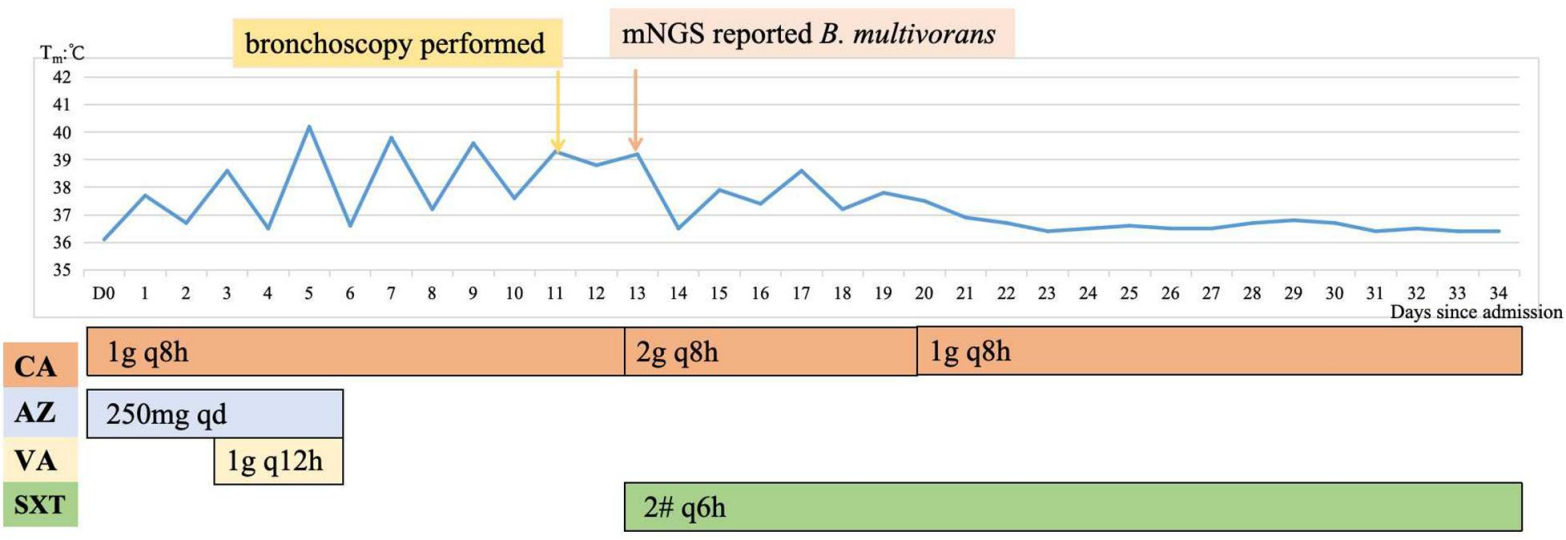
Temperature fluctuations of the patient with a rare compound heterozygote mutation in the CYBA gene as per the treatment adjustments during hospitalization. mNGS, metagenomic next-generation; CAZ, ceftazidime; AZI, azithromycin; VAN, vancomycin; SXT, trimethoprim/sulfamethoxazole; qd, once daily; q6h, four times daily; q8h, three times daily; q12h, twice daily; 2#, two pills of SXT containing 800 mg trimethoprim and 160 mg sulfamethoxazole.

After admission, he received intravenous ceftazidime 1 g every 8 h, azithromycin 250 mg once daily and vancomycin 1 g every 12 h but the fever persisted (Figure 2). Bronchoscopy showed no abnormality, and bronchoalveolar lavage fluid (BALF) cultures were negative. DNA extracted from BALF (QIAamp DNA Microbiome Kit, QIAGEN, Germany) underwent mNGS on an Illumina NovaSeq 6000 (Illumina, San Diego, USA). After filtering low-quality reads and human DNA, *B. multivorans* was identified (34 reads, 35.2% relative abundance, 72.5% genome coverage, and 2.3 ×  depth). This organism was not a background contaminant and was unique to this sample, supporting result reliability. Transbronchial lung biopsy of the left lower lobe showed inflammation accompanied by focal neutrophil infiltration (Figure 1c). Based on these results, antibiotics were switched to oral trimethoprim/sulfamethoxazole (SXT) 800 mg/160 mg four times daily and ceftazidime 2 g every 8 h based on the identified pathogen. His temperature normalized within two days (Figure 2). Repeat thorax CT showed marked resolution of consolidation in the left lower lobe (Figure 1b).

Considering a young age and recurrent episodes of pneumonia, we suspected an inborn error of immunity. Whole exome sequencing (WES) was performed on the patient and his parents. The patient was found to be a compound heterozygote for two mutations in the *CYBA* gene (chromosome 16, exon 1 and 6), which encodes the protein p22*^phox^*(Figure 1d). The mutations were: c.7C > T in exon 1 [Gln3 > Ter], and c.371 C > T in exon 6 [Ala124 > Val] (10, 11). His mother carried the first mutation, and his father the second (Figure 1e). Flow cytometric dihydrorhodamine neutrophil respiratory burst assay, performed according to the published protocol (12), showed severely impaired neutrophil hydrogen peroxide production. To quantify the magnitude of the defect, we calculated the stimulation index (SI) by dividing the mean fluorescence intensity (MFI) of PMA-stimulated neutrophils by that of unstimulated cells. Prior to HSCT, the patient's neutrophil respiratory burst index was 8.17, which is only approximately 2.7% of the normal control value (300.89), indicating a profound defect in NADPH oxidase activity (Figure 1f). No pathogenic or likely pathogenic variants in *STAT3* were identified after careful review. Given the diagnosis of CGD and his high risk of recurrent infections, prophylactic oral antibiotics and itraconazole were prescribed at discharge.

**Figure 3:**
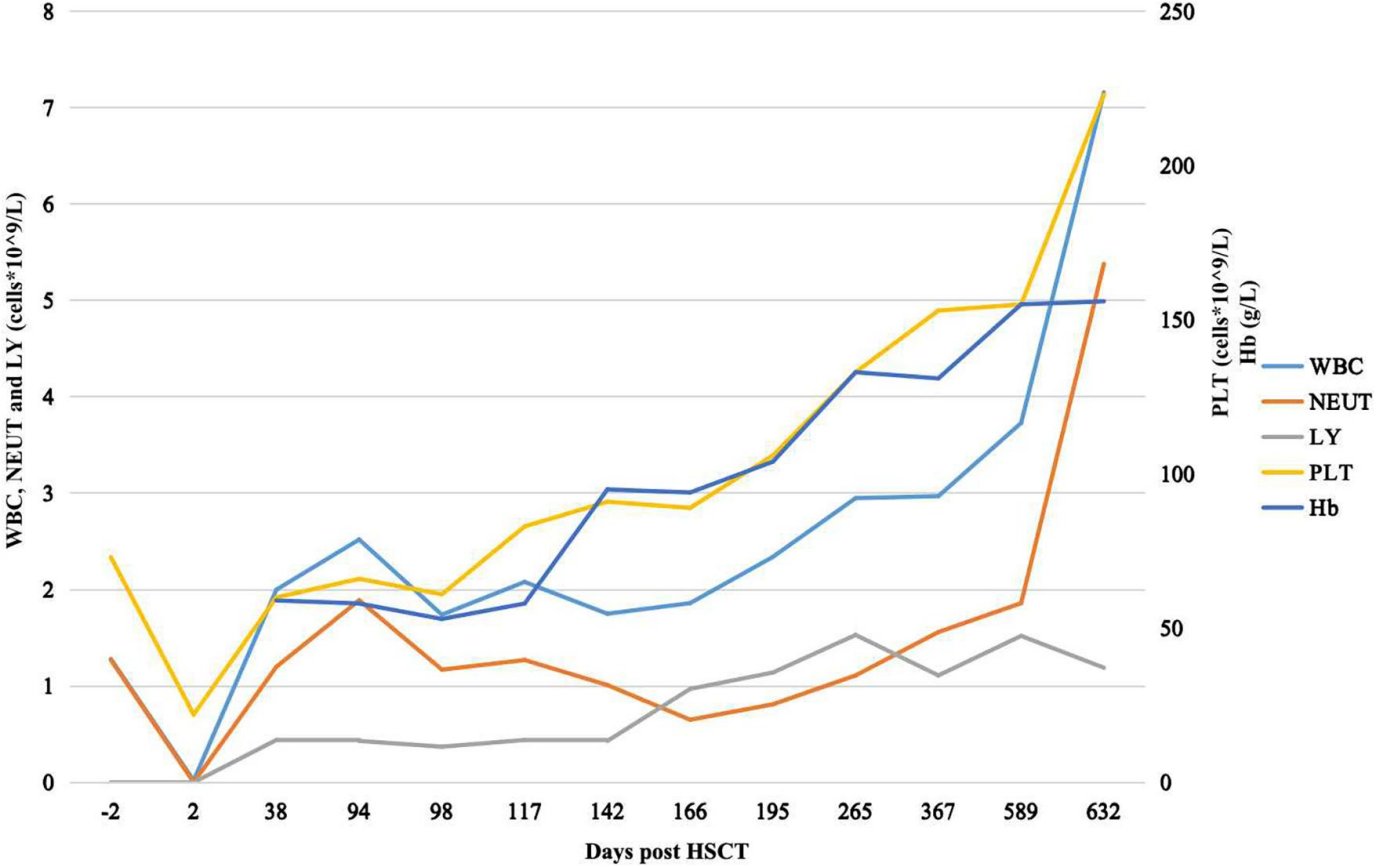
Hematopoietic and immunological reconstitution of the patient with a rare compound heterozygote mutation in the CYBA gene post-HSCT. WBC, white blood cells; NEUT, neutrophils; LY, lymphocytes; PLT, platelets; Hb, hemoglobin.

**Figure 4:**
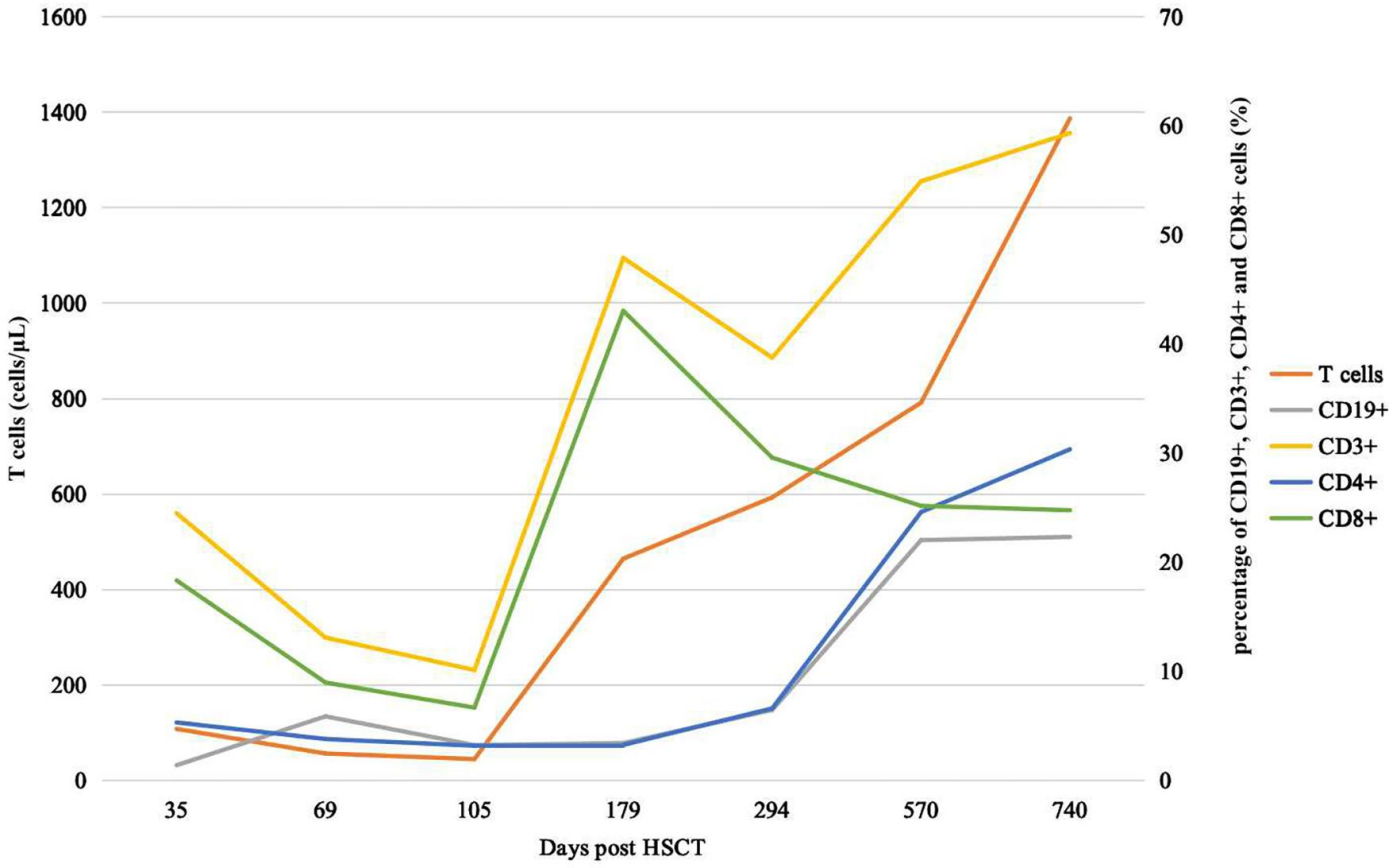
Immunological reconstitution of the patient with a rare compound heterozygote mutation in the CYBA gene post-HSCT.

Despite good adherence to the prophylactic regimen, the patient experienced recurrent fevers upon discontinuation of the medications. Eight months after discharge, the patient underwent allogeneic HSCT from his father, who was a 5/10 HLA-matched donor and ABO incompatible (donor: B positive; recipient: O positive). The transplantation was performed at a specialized center following multidisciplinary discussion and informed consent. Serial assessments of neutrophil function using the dihydrorhodamine respiratory burst assay showed restoration of NADPH oxidase activity to 97.3% at a month post-HSCT and 93.2% at six months. Immunoglobulin (IgG, IgA, IgM and IgE) monitoring, hematopoietic and immunological reconstitution post-HSCT demonstrated expected recovery (Figures 3, 4). Long-term prophylactic medications with antimicrobials and immunosuppressants was carefully tapered based on immune reconstitution: antibacterial prophylactic medications (levofloxacin, isoniazid, SXT) was discontinued 4–16 months post-HSCT, antiviral (acyclovir) and antifungal (itraconazole) prophylactic medications at one year, and ciclosporin at 22 months. Complete withdrawal of prophylactic medications represents a major milestone and a key benefit of curative HSCT, eliminating the lifelong toxicities, costs, and burdens of sequelae. This immune restoration translated into a profound improvement in his quality of life. The patient remained free of CGD-related infections, returned to school and sports, and continued regular clinical and laboratory surveillance. At his most recent evaluation, 25 months post-HSCT and 4 months after stopping all prophylaxis, he remained clinically well, with no relapse or opportunistic infections.

## Discussion and conclusions

CGD is a rare but potentially fatal primary immunodeficiency. The estimated incidence in the USA and Europe is around 1 in 200,000 births, whereas no incidence data are available for China (13). The respiratory burst assay is a key screening tool (14), and abnormal results should be confirmed by genetic analysis. However, in adult-focused hospital settings where the assay is often unavailable, genetic sequencing may be a more practical diagnostic approach, as illustrated by our patient.

Heterozygous and homozygous c.7C > T in exon 1 [Gln3 > Ter] have been reported in patients with CGD in China (10) and Japan (11); homozygous c.371 C > T in exon 6 [Ala124 > Val] has also been reported in Asian and African CGD cohorts (15–17). However, the combination of heterozygous c.7C > T (Gln3 > Ter) and c.371 C > T (Ala124 > Val) in the *CYBA* gene is reported here for the first time. According to the American College of Medical Genetics guidelines, the nonsense mutation *CYBA*: c.7C > T (Gln3 > Ter) can be classified as pathogenic (PVS1 + PM2 + PM3) (11, 18). The variant c.371 C > T (Ala124 > Val) was further analyzed using multiple bioinformatic tools. Conservation analysis shows the alanine at position 124 is highly conserved across mammals. PolyPhen-2 and SIFT predicted the variant c.371 C > T (Ala124 > Val) as “uncertain”, with scores of 0.38 and 0.011, respectively. REVEL and AlphaMissense predicted the variant c.371 C > T (Ala124 > Val) as “deleterious (moderate)”, with scores of 0.83 and 0.97. According to the previous studies (19), REVEL scores can provide PP3 evidence if they meet calibrated thresholds: 0.644–0.772 suggests “supporting pathogenic evidence”; 0.773–0.931 suggests “moderate pathogenic evidence”; and > 0.932 suggests “strong pathogenic evidence”. Thus c.371C > T with a REVEL score of 0.83 qualifies as PP3 Moderate. Taken together, for *CYBA*:c.371C > T, its trans configuration with *CYBA*:c.7C > T supports PM3 evidence, allowing it to be classified as likely pathogenic (PM2 + PP3_Moderate + PM3).

Hyper IgE syndrome (HIES) typically presents with elevated serum IgE, eczematous rash, recurrent pneumonia, and characteristic dysmorphic features such as red-hair, retained primary dentition, a high-arched palate, and recurrent oral candidiasis (20). CGD is characterized by impaired or absent reactive oxygen species production in all phagocytes, predisposing patients to recurrent bacterial and fungal infections (21). Both HIES and CGD can present with recurrent infection. The NIH HIES scoring system, proposed by Grimbacher et al. (22) aids in screening. Scores ≥ 40 points strongly suggest HIES, scores < 20 make HIES diagnosis unlikely, and scores 20–40 are inconclusive. This patient exhibited some HIES-like features, including facial appearance, significantly elevated IgE, and positive *Aspergillus* precipitating IgG, which could be misleading. But he did not exhibit characteristic HIES features such as retained primary teeth, recurrent candidiasis, or skeletal abnormalities. His HIES-NIH score was 29, placing him in the grey zone. The final diagnosis of CGD was confirmed via *CYBA* gene mutations and deficient neutrophil function testing. Similarly, Patiroglu T et al. (23) reported a case of a 9-year-old boy who presented with a characteristic facial appearance, recurrent pneumonia, and elevated IgE levels (HIES-NIH score of 30). Neutrophil function test and molecular analysis revealing a *CYBB* splice site mutation (c.338-1G > A) confirmed X-linked CGD (23). Unlike our patient, that boy was born to consanguineous parents, became symptomatic in the second month of life, and experienced severe infections requiring intensive care, highlighting that patients with autosomal recessive CGD generally have a milder phenotype than X-linked CGD (21).

In this case, mNGS detected *B. multivorans* in BALF, guiding management of his non-resolving pneumonia. *B. multivorans* is rarely isolated in humans compared to other species like *B. cepacia*. A literature review of *B. multivorans* infections was conducted through the Medline/PubMed database. Fourteen studies met the criteria, comprising 12 sporadic cases and 2 outbreaks (Table 2). The age of patients ranged from 1 to 61 years, with no clear sex predominance. *B. multivorans* was most frequently isolated from sputum (7/12, 58.3%), followed by blood (2/12, 16.7%), urine, ascitic fluid, tissue and cerebrospinal fluid, no cases were diagnosed by molecular methods. All but one patient had underlying conditions, most commonly cystic fibrosis. Five patients were immunocompromised, including four post-transplant patients and one hematologic malignancy. Treatment regimens varied (cephalosporins, sulfonamides, carbapenems, and others). Most patients responded well to the treatment though two patients died. Regarding outbreaks, one study reported *B. multivorans* ST1071 contamination of ultrasound gel, leading to bloodstream infections in four pediatric patients in India (24). Another described clonal spread of *B. multivorans* ST439 with poor outcomes — 11 of 46 (24%) infected patients died (25). Literature indicates that *B. cepacia* complex exhibits intrinsic resistance to many antimicrobials, including penicillins, polymyxins, and aminoglycosides, due to efflux pumps (26). SXT, meropenem, doripenem, doxycycline, minocycline, and ceftazidime are among the most active agents (26). SXT combined with an increased dose of ceftazidime successfully controlled his fever and resolved the pneumonia, and he was maintained on long-term SXT and itraconazole to prevent further bacterial and fungal infections (1).

**Table 2:** Review of the literature of Burkholderia multivorans infections.

No.	Year	Location	Age (years)/Sex	Specimen	Co-infection	Clinical manifestaion	Underlying disease	Treatment	Outcome
1	2004	India	53/M	Ascitic fluid	None	Fever, abdominal pain	Hepatitis C, cirrhosis with portal hypertension	Meropenem	Recovered
2	2005	UK	40/F	Sputum	*Streptococcus pneumoniae, Haemophilus influenzae* and *Moraxella catarrhalis*	Chronic cough	Acute bronchitis, mild asthma, mannose binding lectin deficiency	Tobramycin and ceftazidime	Recovered
3	2018	USA	55/M	CSF	None	Fever, chills, nausea, vomiting and abdominal pain	Multiple myeloma	Trimethoprim/sulfamethoxazole	Recovered
4	2018	Sweden	57/M	Urine	None	Dysuria and fever	None	Trimethoprim-sulfamethoxazole	Recovered
5	2019	Canada	23 month/M	Blood, lung tissue	None	Fever	Liver transplant, biliary atresia and Kasai surgery	Cefotaxime, piperacillin/tazobactam, tobramycin, ceftazidime, ciprofloxacin, meropenem, vancomycin and sulfamethoxazole/trimethoprim	Died
6	2022	Spain	61/M	Sputum, bronchoscopic aspirates	None	Not mentioned	Bronchiectasis, lung transplant	Nebulized ceftazidime and inhaled tobramycin	Recovered
7	2006	Germany	30/M	Sputum	None	Not mentioned	Cystic fibrosis	Not mentioned	Not mentioned
8	2011	UK	18/F	Sputum	*Pseudomonas aeruginosa* and *Staphylococcus aureus*	Fever	Cystic fibrosis	Not mentioned	died
9	2013	USA	30/M	Sputum	None	Not mentioned	Cystic fibrosis	Not mentioned	Not mentioned
10	2018	UK	not mentioned	Sputum	*Pseudomonas aeruginosa* and *Candida albicans*	Shortness of breath and cough	Cystic fibrosis	Ceftazidime/avibactam combined with aztreonam	Recovered
11	2019	Italy	32/F	Blood	None	Headache	Cystic fibrosis, lung transplant	Meropenem, trimethoprim-sulfamethoxazole, levofloxacin, ceftazidime/avibactam	Recovered
12	2020	USA	16/F	Sputum	None	Not mentioned	Cystic fibrosis, liver transplant	Meropenem-vaborbactam, nitric oxide	Recovered

CSF, cerebrospinal fluid; M, male; F, female.

HSCT still remains the curative treatment for patients with CGD (27), and risks and benefits must be carefully weighed. Gene therapy for hematopoietic cells (GT-HSC) offers a potential alternative, though it is not yet widely available for routine clinical use (1). Our patient's successful outcome aligns with and extends recent evidence on HSCT for CGD. Chiesa et al. (28) demonstrated that HLA-matched HSCT yields excellent survival, especially in younger patients, while highlighting increased risks with HLA-mismatched donors. In the absence of a fully matched donor, our case reflects the growing role of haploidentical HSCT, as investigated by Riller et al. (29). Their study showed that HLA-haploidentical transplantation is a feasible curative option, with 3-year over survival of 75.9%, despite a notable incidence of graft failure and acute graft-versus-host disease (GVHD) (29). Importantly, our patient did not develop acute or chronic GVHD, underscoring that with careful donor selection, conditioning, and post-transplant management, haploidentical HSCT can yield outcomes approaching those of matched transplants. These findings reinforce that haploidentical HSCT should be considered a viable and potentially curative strategy for patients with CGD lacking HLA-identical donors, particularly when performed early in the disease course.

In conclusion, we present a case of adolescent CGD with a rare compound heterozygous CYBA mutation mimicking HIES, successfully cured by HSCT. The application of mNGS facilitated early identification of *B. multivorans* and timely initiation of appropriate therapy. Because CGD and HIES can share overlapping clinical and laboratory features, early whole-exome sequencing (WES) is crucial for accurate diagnosis.

## Data Availability

The data presented in the study are deposited in the NCBI ClinVa repository, accession number VCV000068212.10 (https://www.ncbi.nlm.nih.gov/clinvar/variation/68212/?term=SCV007595871) and VCV000002264.14 (https://www.ncbi.nlm.nih.gov/clinvar/variation/2264/?term=SCV007596017).
